# Eu^3+^ as a Powerful Structural and Spectroscopic Tool for Glass Photonics †

**DOI:** 10.3390/ma15051847

**Published:** 2022-03-01

**Authors:** Thi Ngoc Lam Tran, Alessandro Chiasera, Anna Lukowiak, Maurizio Ferrari

**Affiliations:** 1 IFN-CNR CSMFO Laboratory and FBK Photonics Unit, Via Alla Cascata 56/C, Povo, 38123 Trento, Italy; maurizio.ferrari@cnr.it; 2 Department of Physics, Politecnico di Milano, Piazza Leonardo da Vinci 32, 20133 Milano, Italy; 3 Department of Materials Technology, Faculty of Applied Science, Ho Chi Minh City University of Technology and Education, Vo Van Ngan Street 1, Thu Duc District, Ho Chi Minh City 720214, Vietnam; 4 Institute of Low Temperature and Structure Research, Polish Academy of Sciences, Okolna 2, 50-422 Wroclaw, Poland

**Keywords:** site-selection spectroscopy, dynamical relaxation, densification process, glass-ceramics, luminescence sensitizers, quantum technologies

## Abstract

The unique properties of the Eu^3+^ ion make it a powerful spectroscopic tool to investigate structure or follow processes and mechanisms in several high-tech application areas such as biology and health, structural engineering, environment monitoring systems and quantum technology, mainly concerning photonics. The traditional method is to exploit the unique photoluminescent properties of Eu^3+^ ions to understand complex dynamical processes and obtain information useful to develop materials with specific characteristics. The objective of this review is to focus on the use of Eu^3+^ optical spectroscopy in some condensed matter issues. After a short presentation of the more significant properties of the Eu^3+^ ion, some examples regarding its use as a probe of the local structure in sol–gel systems are presented. Another section is devoted to dynamical processes such as the important technological role of nanocrystals as rare-earth sensitizers. The appealing effect of the site-selection memory, observed when exciting different sites into the ^5^D_1_ state, which the ^5^D_0_ → ^7^F_0_ emission band reflects following the sites’ distribution, is also mentioned. Finally, a section is devoted to the use of Eu^3+^ in the development of a rare-earth-based platform for quantum technologies.

## 1. Introduction

**Figure 1:**
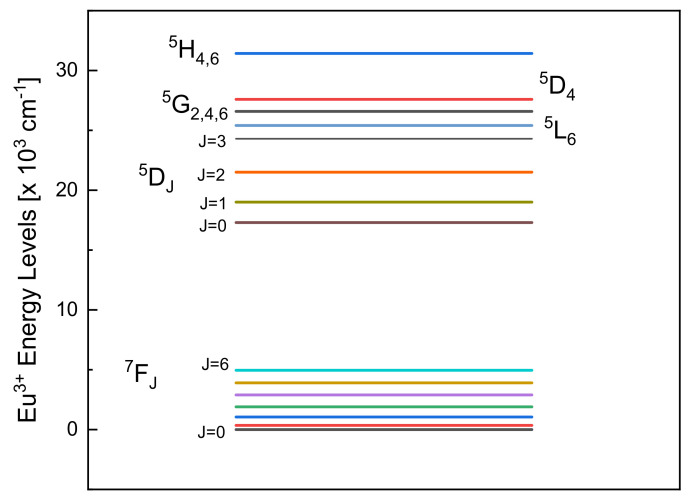
Diagram of Eu^3+^ energy levels often used to probe the local crystal field effect. The energy values were obtained from [5].

Rare-earth ions are characterized by well-defined electronic levels established by the 4*f^n^* electronic configuration [1]. Another important quality, specific to rare-earth ions, is the shielding effect provided by the 5*s* and 5*p* electronic shells to the 4*f* electrons [2]. These properties make rare-earth ions excellent candidates as luminescent probes of the local structure of the system in which they are embedded. Europium trivalent is commonly used because of the clearly defined electric or magnetic dipole character of the transitions among its well-distributed electronic states (Figure 1) [3].

All 4*f*^6^ intraconfigurational transitions are electrical dipoles (ED) forbidden for the isolated ion. When the europium ion is embedded in a matrix, it interacts with the local crystal field. The local symmetry distortion induces a mixing of different parity states, finally allowing electric dipole transitions. Magnetic dipole (MD) transitions are allowed by spin–orbit coupling among different states and, importantly, they are independent, in the first order, from the local symmetry. This means that a comparison of the intensities associated with ED and MD transitions will determine the degree of distortion of the Eu^3+^ site in respect to the centrosymmetric configuration. From this point of view, the ^5^D_0_ → ^7^F_J_ transitions exhibit unique properties. The transition ^5^D_0_ → ^7^F_0_ is a transition between states with J = 0 that is without an internal structure. The transition ^5^D_0_ → ^7^F_1_ is an MD transition, and its intensity should be compared with the ED transition ^5^D_0_ → ^7^F_2_, deemed as hypersensitive due to its effective sensitivity to the local crystal field variations. This asymmetric ratio is a value often employed to compare different matrices or the same matrix with different Eu^3+^ contents or even structural modifications induced by processing [4]. Last but not least, the Stark splitting of the ^7^F_J_ multiplets immediately gives information about the local symmetry, as shown in Table 1 [5].

In this short review paper, we will present some consolidated and novel examples where the spectroscopic properties of the Eu^3+^ ion are employed to obtain information about structures, mechanisms, and processes.

## 2. Eu^3+^ as Probe of Local Structure

### 2.1. OH Coordination Sphere and Densification Process

**Figure 2:**
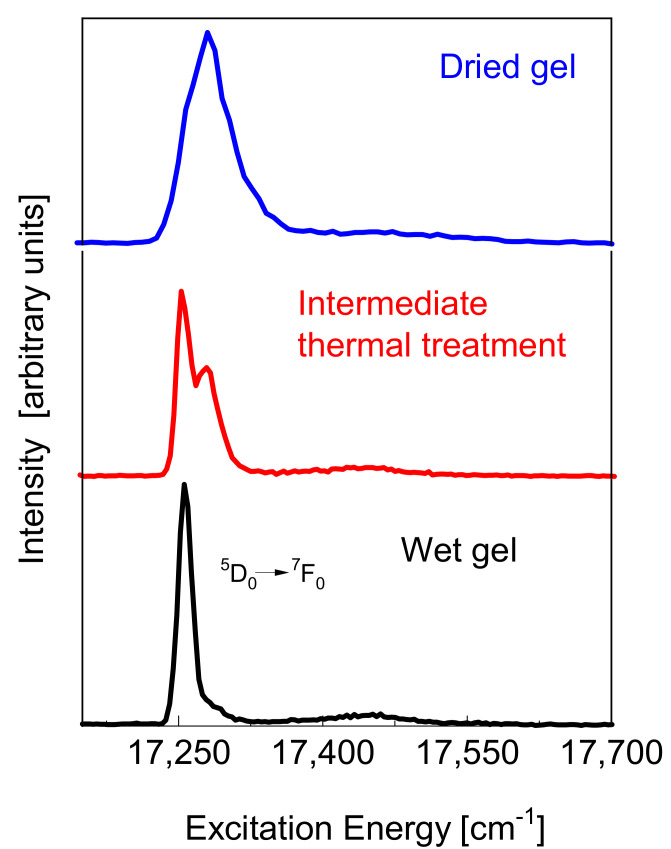
^5^D_0_ → ^7^F_0_ excitation spectra for silica xerogels samples activated by Eu^3+^ ions and heat treated at different temperatures. Obtained by the experimental data concerning the research reported in [11].

It is well known that the presence of OH groups leads to luminescence quenching by non-radiative relaxation due to the O-H stretching vibration with frequency in the range from 3500 to 3900 cm^−1^ [6]. This means that, through lifetime measurements of the ^5^D_0_ state, it is possible to estimate the average number of water molecules in the first coordination sphere of Eu^3+^. Horrocks and Sudnick demonstrated that the non-radiative relaxation rate of the ^5^D_0_ state allowed them to estimate the average number of water molecules coordinated to the Eu^3+^ ion [7]. The average number $N_{H_{2}O}\;$of water molecules can be obtained using the phenomenological equation:

(1) $$N_{H_{2}O}=A_{Ln}/\tau_{OH}$$

where the constant $A_{Ln}$ is typical of the lanthanide ion (in the case of Eu^3+^, $A_{Eu}=1.05\;\mathrm{ms}$ [7]) and the non-radiative decay rate $\tau_{OH}^{-1}$ is due to the O-H vibrations, defined as:

(2) $$\tau_{OH}^{-1}=\tau_{obs}^{-1}-\tau_{R}^{-1}$$

with $\tau_{obs}$ and $\tau_{R}$ equal to the observed lifetime and the radiative lifetime of the ^5^D_0_ state of Eu^3+^, respectively. The radiative lifetime of the ^5^D_0_ state can be estimated by the ratio of the magnetic dipole emission intensity ($I_{MD})$ to the total emission intensity ($I_{Tot})$, as obtained from the luminescence spectra,

(3) $$\frac{\tau^{R}}{\tau_{MD}^{R}}=\frac{W_{MD}}{W_{MD}+W_{ED}}=\frac{I_{MD}}{I_{Tot}}$$

where $\tau_{MD}^{R}$ is the radiative lifetime of MD transition alone and $W_{MD}$ and $W_{ED}$ are the MD and ED transition probabilities, respectively [3,4,8]. We can find a large spectrum of studies where this approach is used. For instance, Popov et al., successfully studied the fluorescence kinetics of the ^4^F_3/2_ level of the Nd^3+^ ion in aqueous colloidal solutions of Nd^3+^: LaF_3_ single-phase crystallized nanoparticles [9]. Aime et al., investigated Ln(III)–malonate complexes with europium in order to assess the possible formation of ternary adducts between the protein and the metal complex [10]. To succeed in this, they employed the proton relaxation enhancement method (PRE), largely used for the determination of the binding parameters when a paramagnetic species interacts with a macromolecule. However, because the PRE method is not suitable when multiple equilibria are present, the authors based the study on the lifetime spectroscopy of Eu^3+^ in a controlled H_2_O and D_2_O environment. Using the approach described above, on the basis of the different numbers of water molecules around the Eu^3+^ ion, the number of paramagnetic species present in solution was determined [10]. There are a huge number of papers in which the unique spectroscopic properties of the Eu^3+^ ion are employed to investigate the densification processes in materials prepared by the sol–gel technique [3,4]. The standard procedure is to collect the luminescence spectra and the lifetimes as a function of the thermal annealing. An interesting example of this is given by Piazza et al., in a paper devoted to the dehydration process in silica gels activated by Eu^3+^ ions [11]. Figure 2 shows the ^5^D_0_ → ^7^F_0_ excitation spectra for silica xerogels samples activated by Eu^3+^ ions and heat treated at different temperatures. The excitation spectra were obtained by detecting the fluorescence at 16,200 cm^−1^, corresponding to the maximum of the ^5^D_0_ → ^7^F_2_ transition. The modification of the ^5^D_0_ → ^7^F_0_ zero-phonon line, as a function of the densification process, is very interesting. The full width half maximum (FWHM) inhomogeneous linewidth is 20 cm^−1^ for the wet gel and 50 cm^−1^ for the dried gel.

**Figure 3:**
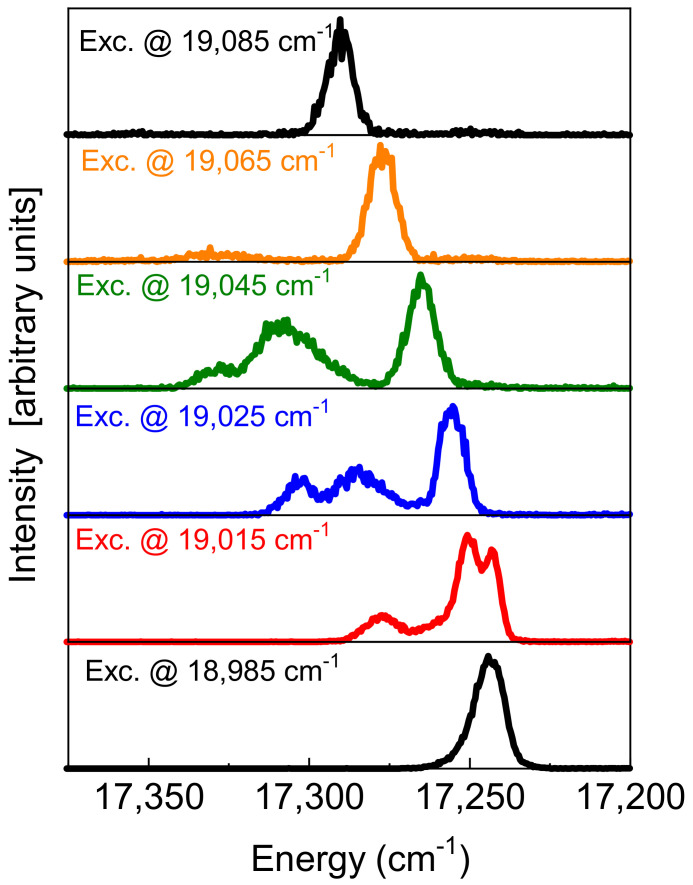
^5^D_0_ → ^7^F_0_ emission spectra for intermediate heat-treated silica xerogel samples activated by Eu^3+^ ions recorded upon excitation at different energies into the ^7^F_0_ → ^5^D_1_ inhomogeneous absorption band. Obtained by the experimental data concerning the research reported in [11].

The densification process, modifying the local environment around the Eu^3+^ ion, was evidenced by the blue shift of the ^5^D_0_ → ^7^F_0_ line. The barycenter is at 17,260 cm^−1^ for the wet gel and at 17,295 cm^−1^ for the dried sample. At intermediate heat treatment, a new component occurs at high energy. Its intensity increases with the thermal treatment. The low-energy band is assigned to a liquid-like environment, and the high-energy one to a dry environment [12]. A weak band appears at about 200 cm^−1^ from the zero-phonon line. This sideband is assigned to the vibrational modes of the europium ion with oxygen ligands. Vibronic sidebands associated with the Eu^3+^ ⟷ O^2^ group have been observed in crystals and glasses [13,14,15]. The sideband is very weak in the wet gel and becomes significant in the dried xerogel. The coordination number (CN) with water molecules remains practically constant during the drying process. A rough estimation, based on Equation (1), gives CN = 7.5 for the wet sample and CN = 6.9 for the sample under intermediate thermal treatment. It could be useful to compare these values with those obtained by X-ray diffraction studies of aqueous rare-earth solutions where the average Eu^3+–^H_2_O distance is 2.450 Å and the average CN is 8.3 [16]. Europium in the wet gel suffers a high symmetric crystal field and the interaction of the ion with the silica network is weak. On these bases a weak covalence is expected. This means that, even in a dry environment, we can have high CN and Eu–O binding lengths not too different from those observed in the liquid, but with stronger electron–phonon coupling [17]. In fact, the formation of the SiO_2_ network and the subsequent local disorder change the binding lengths as well as the magnitude of the covalent interactions between Eu^3+^ and the surrounding oxygen ligands. Piazza et al., obtained other direct evidence of the sidebands by analyzing the excitation spectra in the ^5^D_0_ → ^7^F_0_ and ^5^D_1_ → ^7^F_0_ energy range for the Eu:SiO_2_ xerogels. They observed vibronic sidebands at around 200 and 350 cm^−1^ from the zero-phonon lines in both the excitation spectra. The 350 cm^−1^ band was assigned to an Eu ⟷ O stretching mode, where the interaction is with a non-bridging oxygen of the silica chain [11]. As reported in Table 1, a state with J = 1 splits into three Stark multiplets when the ion is located in a low symmetry site. This is true for the states ^7^F_1_ and ^5^D_1_ of Eu^3+^. Upon selective excitation in the ^5^D_1_ inhomogeneous band of an Eu:SiO_2_ xerogel, Piazza et al., observed a very impressive variation in the ^5^D_0_ → ^7^F_0_ luminescence spectra as a function of the excitation energy. Figure 3 shows the ^5^D_0_ → ^7^F_0_ emission spectra, recorded using different excitations into the ^5^D_1_ state, of the Eu:SiO_2_ intermediate heat treatment. As we discussed above, in the intermediate condition between wet and dry gels, the probability of Eu^3+^ ions not interacting with local crystal field sites is high. Figure 3 shows the interesting effect of the site-selection memory. The site selection, exciting different sites into the ^5^D_1_ state, is reflected by the structure of the corresponding ^5^D_0_ → ^7^F_0_ emission.

It is worth noting that the J = 0 transition presents some internal structures, indicating the presence of different and isolated crystal field sites for the Eu^3+^ ions. Any issue arising from possible spurious overlap with other emissions, such as ^5^D_1_ → ^7^F_0_, is overcome by using an appropriate temporal gate in the emission recording. When the system is fully densified and the network is completely formed, this interesting memory effect is not much more effective due to the energy transfer among Eu^3+^ ions belonging to different sites [3,4,12,18,19,20].

### 2.2. Dynamical Processes

Eu^3+^ has been largely used to demonstrate the spectroscopic dynamics in different optical materials and structures. The role of the Eu^3+^ ion appears to be crucial in several papers concerning the study of energy-transfer processes, luminescence enhancement, optical hybrid filters [21], and thermometric measurements [22]. Eu^3+^ is an excellent ion to develop red phosphors. Lu et al., synthesized a series of red phosphors and studied in detail the variations of fluorescence spectra and lifetimes as a function of the Eu^3+^ concentration and temperature [23]. Based on the asymmetric ratio and the decay curves, these authors determined the optimal concentration and structure for making their product a competitive phosphor with respect to the commercial benchmark [23].

Looking at the temperature effect on the relaxation dynamics of silica xerogels activated by Eu^3+^ ions, there is an interesting paper from Bouajaj et al., discussing the phonon-assisted energy-transfer process [19]. They remarked that, at low temperatures, in densified samples, the linewidth ^5^D_0_ → ^7^F_0_ is narrower for emission spectra than for the ^5^D_0_ ← ^7^F_0_ excitation spectra. Bouajaj et al., measured an FWHM of 55 cm^−l^ for the ^5^D_0_ → ^7^F_0_ emission band and 155 cm^−1^ in the excitation spectrum ^5^D_0_ ← ^7^F_0_. The drastic difference is explained by considering the inhomogeneous nature of the line. This is evident at low temperatures, where the homogeneous contribution is negligible. At room temperature, emission and excitation spectra exhibit the same FWHM [19]. The ^5^D_0_ energy level is strongly dependent on the different strengths and symmetry of the local crystal field suffered by the europium ions. The excitation is redistributed between the ions by a phonon-assisted energy transfer. Bouajaj et al., demonstrated in their paper that the population of the different energetic ^5^D_0_ states follows a Boltzmann thermodynamic law. In fact, experimental spectra were perfectly fitted by the following equation:

(4) $${Г}_{cal}\;\left(E,T\right)={Г}_{exp}\;e^{-\left[\frac{\left(E-E_{0}\right)}{kT}\right]}$$

where ${Г}_{cal}\;\left(E,T\right)$ is the calculated ^5^D_0_ → ^7^F_0_ emission shape at energy *E* and temperature *T*. ${Г}_{exp}$ is the experimental ^5^D_0_ → ^7^F_0_ emission shape weighed by the Boltzmann thermodynamic law. *E*_0_ is the lowest-energy ^5^D_0_ state and its value can be obtained from the experimental excitation spectra [19].

Numerous papers are devoted to the study of the ^5^D_0_ → ^7^F_0_ emission shape as a function of the external conditions. Among these papers, the more impressive are surely those concerning the homogeneous bandwidth Г*_h_* of this transition. Here, we give a brief summary of the physical problem. Glasses are characterized by the structural disorder characteristics of amorphous systems, which are reflected by the inhomogeneous line broadening. The inhomogeneous bandwidth ${Г}_{inh}$ is dependent on the local crystal field and it is practically independent of the temperature. The homogeneous line width ${Г}_{h}$ exhibits a power law dependence on the temperature Г*_h_* ∝ *T^φ^* with φ = 2 at temperatures above 20 K and a linear dependence at very low temperatures [24]. Moreover, the temperature behavior of the homogeneous bandwidth can be considered by looking at the localized modes in the glass [25,26]. Sbetti et al., performed a robust work on measuring the homogeneous bandwidth of the ^5^D_0_ → ^7^F_0_ emission line in a zincborate glass as a function of the temperature range from 20 K to room temperature [25]. The dependence of Г*_h_* was also measured at room temperature upon different excitations across the ^5^D_0_ ← ^7^F_0_ inhomogeneous profile. Sbetti et al., demonstrated a linear increase in Г*_h_* across the ^5^D_0_ ← ^7^F_0_ inhomogeneous profile, evidencing a specific site dependence as mentioned above in the section devoted to the variation of the local environment. This behavior, probably related to the nature of the local vibration, is still not well clarified. Maybe more appealing are the results regarding the dynamical behavior of the Г*_h_* as a function of the temperature. In the whole range of temperatures, they demonstrated a quadratic dependence on the homogeneous bandwidth, confirming the theoretical approach in the description of the optical dephasing typical of the topological disorder [27].

Finally, looking at the dynamical investigation of photonic structures, it is noteworthy that the specific spectroscopic properties of the Eu^3+^ ion were used to assess the waveguiding propagation in dielectric sol–gel-based waveguides. In one of the numerous papers published on this subject, Bhaktha et al., used the red luminescence of Eu^3+^ to probe the waveguiding properties of a novel glass composition and to demonstrate the role of SnO_2_ nanocrystals as rare-earth sensitizers [28]. In this paper, the system of Eu^3+^-doped 75SiO_2_–25SnO_2_ glass-ceramic was thermally processed in order to ensure the homogeneous dispersion of nanocrystals of Eu^3+^:SnO_2_ in a silica matrix [29]. Room temperature excitation spectra were recorded in the range of 280–400 nm, detecting the ^5^D_0_ → ^7^F_2_ Eu^3+^ emission transition. Bhaktha et al., observed that the intensity of the absorption band associated with the SnO_2_ nanocrystals increases by about 15 times, moving from x = 8 to x = 25 mol % in the 1 mol% Eu^3+^-activated (100-*x*)SiO_2_–*x*SnO_2_ samples. This is direct evidence of SnO_2_ nanocrystals as effective rare-earth ion luminescence sensitizers [30].

In the same paper, the authors also demonstrated the structural evolution of 1 mol% Eu^3+^-doped 75SiO_2_–25SnO_2_ glass-ceramic waveguide as a function of the thermal annealing. The first part of the paper focuses on the comparative analysis of the intensities of the ^5^D_0_ → ^7^F_J_ emissions to get a picture of the local environment of the Eu^3+^ ions. The comparison of the emission intensity associated with the ^5^D_0_ → ^7^F_2_ electric dipole transition with the magnetic dipole ^5^D_0_ → ^7^F_1_ emission intensity indicates a progressive decreasing of the asymmetry ratio with the increase in the thermal treatment. In a glass-ceramic 75SiO_2_–25SnO_2_ sol–gel-derived structure, the Eu^3+^ ion is embedded in a less distorted local field with respect to the parent glass. This is a universal method to assess, by optical spectroscopy, whether the nanocrystals characterizing the glass-ceramic systems are activated or not activated by the luminescent species.

The mechanism behind the role of SnO_2_ nanocrystals as effective rare-earth ion luminescence sensitizers in terms of the physics is not yet clear. Eu^3+^ is the tool employed to clarify this specific research. Among other research papers, we mention the work on the site symmetry and host sensitization dependence of Eu^3+^ real-time luminescence in tin dioxide nanoparticles by Cascales et al. [31]. The authors made two important dynamical aspects clear. As an outcome of the research, they presented a structural model explaining the role of the oxygen vacancies in exciting the Eu^3+^ ion by direct SnO_2_ nanocrystal excitation. They gave direct experimental evidence of this process by investigating the real-time spectro-temporal dynamics of the SnO_2_ nanocrystals and the Eu^3+^ emissions obtained by multiphoton bandgap pumping by using ultrafast spectroscopy [31].

## 3. Eu^3+^ in Quantum Technologies

Rare-earth ions appear to be an important platform as hardware for quantum technologies [32]. It has already been demonstrated that oxide nanocrystals, when activated by rare-earth ions, exhibit a coherence lifetime in the order of tens of microseconds. The main research in this field is driven by groups that have published some crucial papers on this topic [33,34,35,36,37].

Coherent processes in atomic ensembles are extensively investigated for quantum memory, atomic populations management, and interactions among optical fields. Electromagnetically induced transparency (EIT) is fundamental for several applications, including optical quantum memory, quantum sensing, and electromagnetic shields, among others. The majority of the EIT experiments and other coherent processes in atomic ensembles have been conducted in gaseous environments. Solid-state systems offer specific advantages with respect to gaseous systems. These advantages include higher densities, absence of motion-induced dephasing, and the technological compatibility with integrated photonics architectures. Moreover, rare-earth atoms embedded in solid structures are effective systems for quantum applications due to their particularly long spin coherence times and sufficiently reduced spectral diffusion. However, as discussed above in Section 2.2, the intrinsic difficulty in working with solid-state systems is the static inhomogeneity due to the site dependence of both optical and spin transitions. The effect of this inhomogeneous broadening on the coherence processes has been studied by looking at Doppler broadening in gasses. In solids, the motion-induced dephasing can be neglected so that the coherence could be unconstrained by the transit time effect of atoms through the optical field. Recently, Haoquan Fan and Elizabeth A. Goldschmidt presented an exciting experiment where a solid-state system, based on europium-doped yttrium orthosilicate, was employed to study Λ-type EIT [38]. They exploited the inhomogeneous broadening of the optical and spin transitions of the Eu^3+^ ion building, a so-called Λ-type energy level scheme, as described in [39]. Fan and Goldsmidt performed spectral hole burning measurements at very a high spectral resolution in Eu^3+^:Y_2_SiO_5_ crystals [38]. Eu^3+^:Y_2_SiO_5_ presents three hyperfine ground states and three hyperfine excited states. These nine transitions are dipole allowed with different oscillator strengths. By applying a specific frequency, the authors selected a single optical frequency class of the Eu atoms and manipulated the inhomogeneous width of the final spectrum. To summarize, the authors observed narrowband EIT in a solid-state ensemble of europium atoms over a range of coupling and inhomogeneity parameters. There is an enormous interest in understanding coherent optical processes in inhomogeneously broadened solids, and such systems are considered the more suitable platforms for quantum optics and quantum information.

**Figure 4:**
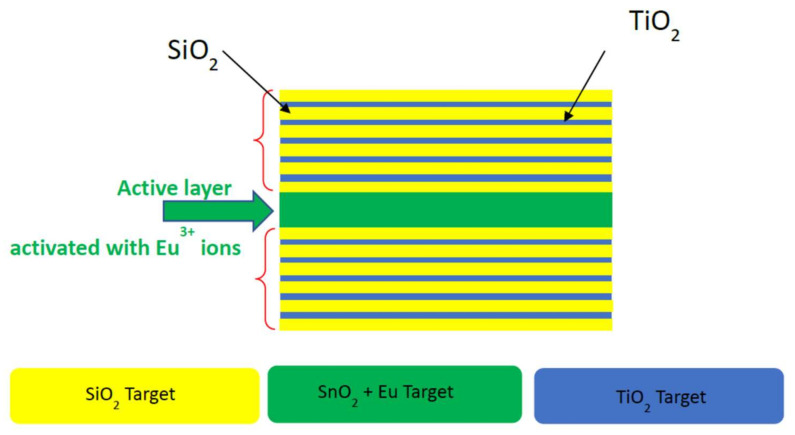
Schematic structure of a Fabry–Perot microcavity that could be developed to make the ion redout effective.

We conclude this section by mentioning that, on the basis of the consolidated skills and experimental results mentioned above, researchers are exploiting the properties of 1-D microcavities to manipulate the inhomogeneous and homogeneous linewidths of the ^5^D_0_ → ^7^F_0_ transition of Eu^3+^ in nanocrystal [32,40]. Figure 4 shows a schematic structure of a Fabry–Perot microcavity that could be developed to make the ion redout effective. The two Bragg structures are constituted by SiO_2_ and TiO_2_ as low and high refractive index components, respectively. The active layer is constituted by Eu^3+^-activated SnO_2_ nanocrystals. For instance, in the case of SnO_2_, its role is to allow a tailored inhomogeneous distribution playing on the local crystal field instead of on the nanocrystals size. In fact, in the SnO_2_ nanocrystal, Eu^3+^ is substitutional for Sn^4+^ with related local field modifications modulated by charge compensation [29].

Moreover, the SnO_2_ nanocrystal band gap absorption at 340 nm maintains the resonance and the pumping schema far from the other when we do not want interference with the cavity band gap. The cavity, when resonant with the rare-earth ion transition, constitutes a scalable device to obtain high redout reliability. Looking at the coherence lifetime, we point out that Eu^3+^ is a perfect ion because of its simple structure of energy levels and the well-defined character of the transitions. TiO_2_ has low and high refractive index components, respectively. The active layer is constituted by Eu^3+^ activated SnO_2_ nanocrystals.

This is a very hot topic, reflected by the ambitious projects and novel architectures proposed in the recently upgraded literature. What is certain is that rare-earth ions in confined structures, and in particular Eu^3+^, are recognized as crucial platforms for quantum computing [32].

## 4. Conclusions

In this brief review, we have highlighted some specific examples where the Eu^3+^ is used to probe structural properties and dynamical processes, including the very new topic of quantum technologies. The unique properties of the trivalent europium were presented, making clear the relation between the degeneracy of the optical transition and the local symmetry. In the same section, the concept of the asymmetric ratio and the electric dipole and magnetic dipole character of the more commonly used transitions were presented. The first example of the use of the Eu^3+^ as a probe of the static local crystal field was the study of OH coordination sphere and densification process in sol–gel-derived systems. The second example discussed the densification process and the use of the evolution of the ^5^D_0_ → ^7^F_0_ zero-phonon line. The FWHM inhomogeneous linewidth modification at different heat treatments was discussed and the presence of different local sites was demonstrated. Of particular interest is the direct evidence of the vibronic sidebands in Eu^3+^:SiO_2_ xerogels. In the second part, devoted to the dynamical processes, the drastic difference observed at low temperature between the linewidth ^5^D_0_ → ^7^F_0_ for emission spectra and the ^5^D_0_ ← ^7^F_0_ excitation spectra was explained considering the inhomogeneous nature of the line. The ^5^D_0_ energy level is site dependent and, at room temperature, the excitation is redistributed between the ions by a phonon-assisted energy transfer. The other dynamical issue discussed in this review concerns the structural evolution of the 1 mol% Eu^3+^-doped 75SiO_2_–25SnO_2_ glass-ceramic waveguide as a function of the thermal annealing and the evidence of the effective role of SnO_2_ nanocrystals as luminescence sensitizers. Finally, we closed the review with some comments concerning the exploitation of Eu^3+^ in quantum technologies. On the basis of the homogeneous and inhomogeneous characters of the transitions, we reported an example in which narrowband EIT is observed in a solid-state ensemble of europium atoms over a range of coupling and inhomogeneity parameters. Still considering the enormous interest in developing suitable platforms for quantum optics and quantum information, we discussed the possibility of developing glass-derived 1-D microcavities to manipulate the inhomogeneous and homogeneous linewidths of the ^5^D_0_ → ^7^F_0_ transition of Eu in nanocrystals.

In conclusion, this short review demonstrates that Eu^3+^ spectroscopy is still powerful enough to investigate new materials and new physics.

## Figures and Tables

**Table 1:** Stark-level splitting for specific crystal field symmetries as a function of the total angular momentum J.

Local Symmetry	Triclinic (C_1_, C_i_)	Monoclinic (C_s_, C_2_, C_2h_)	Rhombic (C_2v_, D_2_, D_2h_)
J = 0	1	1	1
J = 1	3	3	3
J = 2	5	5	5
J = 3	7	7	7

## Data Availability

Not applicable.
